# Therapeutic Potential of Glutamine Pathway in Lung Cancer

**DOI:** 10.3389/fonc.2021.835141

**Published:** 2022-02-11

**Authors:** Enyu Tang, Siyang Liu, Zhiming Zhang, Rixin Zhang, Dejing Huang, Tong Gao, Tianze Zhang, Guangquan Xu

**Affiliations:** Department of Thoracic Surgery, Second Affiliated Hospital of Harbin Medical University, Harbin, China

**Keywords:** metabolic reprogramming, glutamine, lung cancer, target, glutamine transporter, glutaminase

## Abstract

Cancer cells tend to obtain the substances needed for their development depending on altering metabolic characteristics. Among the reorganized metabolic pathways, Glutamine pathway, reprogrammed to be involved in the physiological process including energy supply, biosynthesis and redox homeostasis, occupies an irreplaceable role in tumor cells and has become a hot topic in recent years. Lung cancer currently maintains a high morbidity and mortality rate among all types of tumors and has been a health challenge that researchers have longed to overcome. Therefore, this study aimed to clarify the essential role of glutamine pathway played in the metabolism of lung cancer and its potential therapeutic value in the interventions of lung cancer.

## Introduction

For nearly a century, tumors have been found to display metabolic activities that distinguish them from well-differentiated, non-proliferative tissues, which may contribute to their physiological survival and growth (1). The study of cancer metabolism can thus reveal fundamental aspects of malignancy and has the translational potential to improve the way we diagnose, monitor, and treat cancer. Cancer patients have been summarized as having several metabolic features, including deregulated uptake of glucose and amino acids, opportunistic patterns of nutrient acquisition, utilization of glycolytic/tricarboxylic acid cycle intermediates for biosynthesis and reduced nicotinamide adenine dinucleotide phosphate (NADPH) production, increased nitrogen requirements, altered metabolite-driven gene regulation, and metabolic communication with the microenvironment (2). With the help of these metabolic alterations, tumor cells can meet their own needs for progression and adapt to changes in the tumor environment.

Glutamine (Gln) is one of the most abundant amino acids in the human body, and its importance as cancer nutrient stems from its ability to contribute its nitrogen and carbon to a range of growth-promoting pathways (3). Gln is imported *via* transporters, and its catabolism begins with conversion to glutamate; these reactions either provide amide nitrogen to biosynthetic pathways or release it in the form of ammonia. The latter reaction is catalyzed by glutaminase (GLS), which also generates glutamate (4). On the one hand, glutamate is a major source of α-ketoglutarate (α-KG), which plays a significant role in supplying energy and biosynthesis; on the other hand, glutamate is also a precursor of the major cellular antioxidant glutathione, which is used to maintain intracellular redox homeostasis (5, 6). However, Gln metabolism is altered in the cancer setting in different pathways to respond to the metabolic needs of cancer cells, including imbalance in energy regulation, sustaining proliferative signaling, induction of replicative immortality, inhibition of cell death, and promotion of invasive metastasis (3). Although glucose *via* glycolysis is usually used as the first sequence of energy supply, proliferating cancer cells also rely on glutamine as the primary source of energy and building blocks. They exhibit a dependence on other nutrients to feed the tricarboxylic acid (TCA) cycle, which is typically glutamine. This condition is known as glutamine addiction (7). Glutamine metabolism can interact with glycolysis in surprising ways. Indeed, upregulation of lactate, which may be present in the microenvironment as a consequence of increased glycolysis in cancer cells, has been shown to promote glutamine metabolism through a HIF2- and MYC-dependent mechanism, potentially providing a way for tumor to “reprogramme” itself towards increased glutaminolysis (8). Since glutamine-derived α-KG fuels the TCA cycle, cancer cells can utilize glutamine catabolism to sustain the biosynthesis of many essential molecules. For example, cancer cells can rely on the reductive carboxylation of glutamine-derived citrate to produce acetyl coenzyme A (acetyl-CoA) and other precursors of TCA cycle metabolites (7). Furthermore, glutamine plays a key role as a precursor to other anabolic processes, such as the pentose phosphate pathway (PPP), which produces reduced nicotinamide adenine dinucleotide phosphate (NADPH) (9). Conversely, NADPH generated by the pentose phosphate pathway and cancer-specific serine glycolytic diversion, appears to maintain glutamine utilization for amino-acid synthesis, lipid synthesis and reactive oxygen species (ROS) quenching (10).

**Figure 1:**
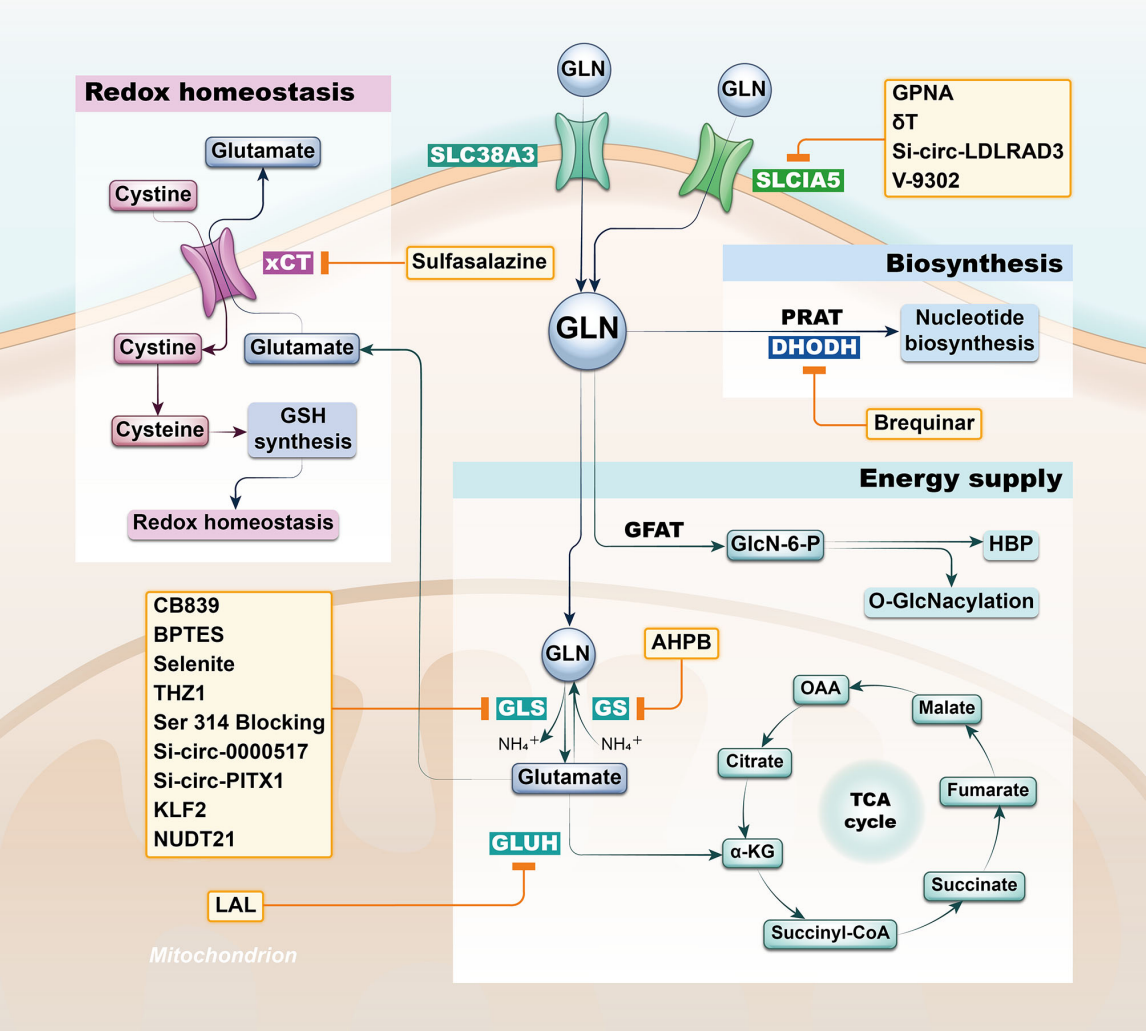
Strategies based on glutamine metabolism. The glutamine pathway is involved in a variety of functional metabolic activities in lung cancer cells, including energy supply, biosynthesis and redox homeostasis, and researchers have developed potential therapeutic strategy by targeting the glutamine pathway based on differential metabolic characteristics of tumor cells. SLC1A5, solute carrier family A1 member 5; GLN, glutamine; GFAT, Gln-fructose-6-phosphate aminotransferase; GLS, glutaminase; GS, Gln synthetase; GLUH, glutamate dehydrogenase; PRAT, phosphoribosyl pyrophosphate amidotransferase; DHODH, dihydroorotate dehydrogenase; xCT, solute carrier family member 7A11.

Intermediates from Gln pathway in tissue, serum, urine, and bronchoalveolar lavage fluid from both lung cancer (LC) and healthy patients analyzed by non-targeted metabolomics are significantly different and proved to be associated with the development of lung cancer (11–14). Not only can the Gln pathway be helpful to the diagnosis of lung cancer patients, but in combination with clinical patient data, its intermediates can also be used as a predictor of overall survival in lung cancer patients (15–18). Actually, there was also variability in the expression of the glutamine pathway between lung cancer subtypes. It’s found that Lung adenocarcinoma and lung squamous cell carcinoma have differential presentation; while adenocarcinomas were found to have higher levels of solute carrier family A1 member 5 (SLC1A5) and GLS in both protein and mRNA levels, squamous lung cancers had higher glucose transporter 1 (GLUT1) expression compared to lung adenocarcinomas. Furthermore, SLC1A5 protein expression was significantly higher in lung adenocarcinomas with lymph node metastasis compared to node negative tumors, as well as increased in adenocarcinomas with higher pTNM staging (19). In recent years, researchers have developed potential therapeutic strategies to target Gln metabolism based on the unique characteristics of Gln metabolism in oncology patients (20). Lung cancer is the consistently leading cause of cancer death with limited treatment options; thus, we purposed to elaborate the potential therapeutic value of Gln pathway in lung cancer from different perspectives with the aim of contributing to the future diagnosis and management of lung cancer patients (**Figure 1** and **Table 1**).

**Table 1:** Therapeutic strategies based on glutamine metabolism.

Target	Agent/strategy	Mechanism	Remark	Reference
SLC1A5	GPNA	Inducing apoptosis and oxidative stress by inhibiting SLC1A5	Animal experiments and NSCLC cell lines (A549, HCC15, etc.)	(21)
	v-9302	Pharmacological blockade of SLC1A5	Animal experiments and lung cancer cell lines (A549, H23, etc.)	(22)
	δT	Inhibiting cell proliferation and inducing apoptosis *via* downregulation of the mTOR pathway	NSCLC cell lines (A549 and H1299)	(23)
	Si-Circ-LDLRAD3	Overexpressing miR-137 by knock-down of circ-LDLRAD3	NSCLC cell lines (A549, Hcc827, etc.)	(24)
GLS	CB-839	A potent reversible noncompetitive allosteric GLS inhibitor	Animal experiments and clinical trial	(25)
	THZ1	Altering the expression pattern of GLS isoforms through deregulation of NUDT21.	NSCLC cell lines (H23, H1299, etc.)	(26)
	Phospho-Ser314 blockade	Blocking GAC phosphorylation through NF-κB-PKCϵ axis	NSCLC cell lines (H23, H1299, etc.) and Human bronchial epithelial cell line	(27)
	Si-circ_0000517	Suppressing proliferation by modulating miR-330-5p/YY1 signal pathway	Animal experiments, Human bronchial epithelial cell line HBE1 and NSCLC cell lines (A549, H1299, etc.)	(28)
	Si-Circ-PITX1	Inhibiting progression through regulating the miR-1248/CCND2 axis	Animal experiments and NSCLC cell lines (HCC827 and H1650)	(29)
	BPTES	Reducing thymidine and carbamoyl-phosphate synthesis	NSCLC cell lines (A549)	(30)
	Selenite	Inhibiting glutaminolysis and glutathione synthesis by suppressing GLS expression	NSCLC cell lines (A549)	(31)
	KLF2	Inhibiting the energy metabolism	NSCLC cell lines (A549, NCI-H1299)	(32)
GS	AHPB	A structural analog of glutamate	NSCLC cell lines (A549)	(33)
xCT	Sulfasalazine	Decreasing the supply of cystine	Animal experiments, NSCLC cell lines (A549, etc.), SCLC cell lines (HCC59, etc.), H460 and H727.	(34)
Glutamine	PGS	Drug or gene-editing molecules delivery system	Animal experiments, Human cervix epithelial carcinoma cells (Hela), human hepatoblastoma cells (HepG2), etc.	(35)
	GPI	Drug or gene-editing molecules delivery system	Animal experiments and NSCLC cell lines (A549)	(36)
	PGG-PTX	Drug or gene-editing molecules delivery system	Animal experiments and NSCLC cell lines (A549)	(37)

## Tumor Suppression by Targeting the Intermediates in the Gln Pathway

### Gln Transporter

Gln firstly enters cells *via* Gln transporters such as solute carrier family A1 member 5 (SLC1A5), which is essential for cancer cell growth (38). SLC1A5 enhances the uptake of Gln in the tumor microenvironment, which in turn enhances cell proliferation and migration by increasing phosphorylation of the mTOR complex 1 signaling axis; subsequently, overexpression of SLC1A5 is found to be relevant to patient prognosis and can act as a separate prognostic factor in patients with lymph node metastases combining with clinical information (39–42). The targeting of SLC1A5 can be performed by RNA interference and a small molecule inhibitor gamma-L-glutamyl-p-nitrosamides (GPNA) in non-small cell lung cancer (NSCLC) and human bronchial cell lines. In a subpopulation of NSCLC cell lines overexpressing SLC1A5, inactivating SLC1A5 either genetically or pharmacologically could reduce Gln consumption, inhibit cell growth, and induce its autophagy and apoptosis. Targeting SLC1A5 in *in vivo* experiments also reduced tumor growth in NSCLC xenografts (21). V-9302 is another small molecule antagonist of transmembrane glutamine flux that competitively and potently targets the glutamine transporter SLC1A5. It’s verified that pharmacological blockade with V-9302 could result in attenuated cancer cell growth and proliferation, increased cell death and oxidative stress in lung cancer cell (22). Delta-tocotrienol (δT) treatment could similarly suppress the activity of SLC1A5 by derivatizing glutamate and glutathione, as well as some essential amino acids; while knockdown of circ-LDLRAD3 reduced the expression of SLC1A5 by sponging miR-137, which was eventually also observed to inhibit the development of NSCLC (23, 24). SLC38A3, another transporter of Gln, activates the PDK1/AKT signaling pathway and promotes the metastasis of NSCLC by regulating the transport of Gln and histidine, indicating that SLC38A3 owns consistent therapeutic potential for the treatment of NSCLC (43). In addition to the above glutamine transporters that have been shown to have therapeutic potential in lung cancer cell lines, researchers have also identified other glutamine transporters in other cancer cell lines, which might also be involved in the development of lung cancer (44, 45). It was found that sodium neutral amino acid transporters (SLC38A1/2) and L-type amino acid transporters (LAT1/SLC7A5) could be inhibited by 2-amino-4-bis(aryloxybenzyl)aminobutanoic acids (AABA) upon SLC1A5 depletion in osteosarcoma cells and breast cancer cells, thereby suppressing cancer progression (46). This finding, although not validated, may also has similar effects on lung cancer cells and merits further exploration in the future.

### Glutaminase

Cellular utilization of Gln is generally initiated by conversion of Gln to glutamate, and inhibition of Glutaminase (GLS) in tumor cells which develop dependent on Gln metabolism deprives cells of glutamate, leading to disruption of tumor metabolic pathways including: macromolecular synthesis, ATP production, and cellular redox homeostasis. The conversion of Gln to ketoglutarate, a tricarboxylic acid cycle intermediate, *via* glutaminase and alanine aminotransferase, is critical for tumor development, and thus targeted therapy by selective inhibition of GLS is a novel strategy of considerable interest for the treatment of cancer.

CB-839, a potent reversible noncompetitive allosteric GLS inhibitor, in combination with erlotinib for EGFR-mutated non-small cell lung cancer, inhibits tumor growth by impairing both glucose and Gln utilization (25). The application of CB839 in lung cancer has actually been validated in clinical trials, of which NCT02771626 and NCT02071862 have been completed but results are not yet available, while NCT03831932, NCT04250545 and NCT04265534 are in progress. Many NSCLC cell lines differ in their sensitivity to glutaminase inhibitors, making it particularly important to determine the metabolic profile under which they can be efficacious against cancer cells. Alanine-derived pyruvate catabolism induced by alanine transaminase can promote mitochondrial metabolism through the production of acetyl coenzyme A (acetyl-CoA) in the absence of Gln-derived alanine and ketone glutaric acid, therefore driving NSCLC cell survival after glutaminase inhibition (47). Alanine is the second most abundant circulating amino acid after Gln, reaching plasma micromolar concentrations, thus serving as an important metabolic mechanism to overcome resistance to CB-839.

GLS can exist as several splice variants by selective splicing, and KGA is considered to be full-length GLS. KGA is derived from exons 1-14 and 16-19, while a second GLS splice variant, glutaminase C (GAC), has another carboxy terminus (from exon 15 only), resulting in a lower molecular weight protein (48). Expression of the GLS splice variant KGA was reduced in tumors compared to normal lung tissue. Transient knockdown of the GLS splice variant indicated that deletion of GAC had the greatest effect on cancer cell growth (49). HIF-1α-mediated downregulation of NUDT21 under hypoxic conditions altered the expression patterns of both isoforms of GLS, GAC and KGA. These results link the tumor hypoxic environment to aberrant Gln metabolism, which is important for the cellular energy supply of small cell lung cancer (SCLC) cells; therefore, NUDT21 can be considered as a potential target for SCLC therapy (50). THZ1, a covalent inhibitor of cell cycle protein-dependent kinase 7 (CDK7), can alter the expression pattern of glutaminase isoforms by promoting ubiquitination and degradation of NUDT21, ultimately inhibiting the proliferation and migration of lung cancer cell lines (26). The Ser314 phosphorylation site is an important post-translational modification site of GAC, which is regulated by the NF-κB-PKCϵ axis. Besides, it was evidenced that high level of GAC phosphorylation is associated with low survival in lung cancer patients, meanwhile blocking Ser314 phosphorylation in lung cancer cells inhibits glutaminase activity and triggers gene reprogramming, subsequently suppressing lung cancer progression (27).

Circular RNA (circRNA) is a sort of non-coding RNA with a circular structure that is widely and stably expressed in eukaryotic cells. CircRNA has shown promise in becoming a biomarker for human diseases, especially cancer, attributing to its abundance and stability. Circ_0000517 was highly expressed in lung cancer tissues and cells, while circ_ 0000517 overexpression was negatively correlated with the prognosis of NSCLC patients. Circ_0000517 silencing significantly inhibited proliferation, glycolysis and Gln catabolism in NSCLC cells and suppressed xenograft tumor growth. Silencing circ_0000517 shrunk SLC1A5 and GLS expression by regulating miR-330-5p/YY1 axis and ultimately improved NSCLC progression (28). In addition, circ-PITX1 expression is upregulated in NSCLC, and its silencing inhibits GLS expression through the miR-1248-CCND2 pathway, thereby inhibiting Gln metabolism in NSCLC cells, promoting NSCLC cell apoptosis and suppressing tumor growth *in vivo* (29).

Studies dedicated to targeted therapeutic potential by selective inhibition of GLS have yet other different perspectives. The use of bis-2-(5-phenylacetamido-1,3,4-thiadiazol-2-yl)ethyl sulfifide (BPTES) can reduce metabolic intermediates such as thymine and carbamoyl phosphate by inhibiting GLS, which additionally reduces pyrimidine supply with the concomitant use of 5-FU in NSCLC, thereby synergistically inducing cell death (30). Ronald C. Bruntz et al. found that selenite inhibited Gln catabolism and glutathione synthesis by suppressing GLS expression but transiently activated pyruvate carboxylase activity, afterwards supplementation with glutamate partially rescued these antiproliferative and oxidative stress activities. Similar metabolic perturbations and cell necrosis were evident in cancer tissue interfered by selenite in *in vivo* experiments, suggesting that selenite is also a potential therapeutic direction to affect lung carcinogenesis through targeting GLS (31). Kruppel-like factor 2 also can inhibit glutaminase expression, thereby suppressing energy metabolism and proliferation of NSCLC cells (32). In summary, glutaminase is a major hot point in the application of Gln metabolic pathway in lung cancer research, and although it has been favored by the majority of researchers, there is still some distance away from practical application in patient diagnosis and treatment.

### Nucleotide Biosynthesis

Much attention has been paid to the inhibition of GLS in current studies focusing on targeting Gln metabolism in lung cancer patients. Actually, GLS expression levels are positively correlated with extracellular Gln concentrations; therefore, GLS is highly expressed in environments containing high concentrations of Gln (51). However, the low levels of extracellular Gln to which cancer cells are exposed *in vivo* may maintain GLS expression at a low standard, thereby limiting the sensitivity of cells to its inhibitor (52). Proteomic and metabolomic data of Manabu Kodama et al. consistently indicate that the balance of Gln utilization appears to be tilted from Gln catabolism toward nucleotide biosynthesis during malignant progression (53). For *de novo* purine nucleotide biosynthesis, glutamine-derived nitrogen is transferred to phosphoribosylpyrophosphate (PRPP), a nucleic acid precursor generated by the pentose phosphate pathway (PPP), in a rate-limiting reaction mediated by phosphoribosyl pyrophosphate amidotransferase (PRAT) (54). Glutamine-derived nitrogen is also essential for the rate-limiting synthetic reaction of carbamoyl phosphate (CP), a precursor to pyrimidine nucleotides (55). Thus, glutamine is indispensable as a nitrogen donor in anabolic reactions in the *de novo* nucleotide biosynthesis pathway. Indeed, the strong correlation between the expression of PRAT and poor prognosis in patients with small cell lung cancer and the inhibition of non-anchored growth of SCLC cell lines by PRAT deficiency suggest that PRAT may be a promising therapeutic target for SCLC. Furthermore, inhibition of the transfer of glutamine-derived nitrogen to the *de novo* nucleotide biosynthesis pathway by forced expression of PRAT-specific short hairpin RNA significantly inhibited the proliferation of SCLC cell lines (53). Given that PRAT appears to be one of the most pro-malignant of all metabolic enzymes in human cancer, the search for inhibitors of PRAT warrants further efforts. Researchers have also identified disruptors that target the *de novo* synthesis of pyrimidine nucleotides. Brequinar, an inhibitor of dihydroorotate dehydrogenase (DHODH) in the *de novo* pyrimidine biosynthesis pathway, was found to suppress tumor growth in xenograft models of SCLC (56). In conclusion, glutamine is essential for nucleotide synthesis in cancer cells, and targeting the glutamine pathway would therefore significantly hinder the development of cancer cells.

### Gln-Fructose-6-Phosphate Aminotransferase

Gln-fructose-6-phosphate aminotransferase (GFAT) is the rate-limiting enzyme of the hexosamine biosynthetic pathway (HBP), and it will transfer amide nitrogen from Gln to fructose-6-phosphate in the first and subsequently generate amine-6-glucose phosphate (GlcN-6-P). GlcN-6-P is acetylated to N-acetylglucosamine-6-P, which is then converted to N-acetylglucosamine-1-P by a mutase reaction, and it reacts with UTP to form UDP-n acetylglucosamine in the end, which is the final product of the pathway, providing substrates for post-translational modifications of the nucleus, cytoplasm, and mitochondrial proteins (57). O-GlcNAcylation is a key regulator of a variety of cellular processes, such as signal transduction, transcriptional regulation, and proteasomal degradation. Elevated expression of O-GlcNAc transferase and O-GlcNAc has been observed in various types of cancer and has been found to promote cancer growth and progression (58). A study by Weiruo Zhang et al. found elevated expression of a few related genes encoding Gln-fructose-6-phosphate aminotransferase in lung adenocarcinoma (AD) and identified GFAT as a key regulator of metabolic reprogramming in AD (59). Metabolomics and gene expression profiling showed that the hexosamine biosynthesis pathway (HBP) was activated in mouse and human KL (*KRAS/LKB1* cotransformant) mutant tumors, and that KL cells contained high levels of HBP metabolites, had higher flux through the HBP pathway, and were more dependent on the HBP rate-limiting enzyme GFAT. Inhibition of GFAT selectively reduced the growth of KL tumor cells in cultured cells, xenografts and transgenic mice (60). Additionally, changes in GFAT activity were also observed following chemical treatment of cancer cells. Cisplatin inhibits AMP-activated protein kinase (AMPK) activity by decreasing the AMP/ATP ratio. However, the decrease in AMPK activation inhibited the phosphorylation of GFAT, which in turn promoted GFAT activity and ultimately upregulated the expression of O-GlcNAc transferase and O-GlcNAcase and led to the increased levels of global protein O-GlcNAc in H1299, HepG2 and MCF7 cells (61). PD-L1 can be involved in the regulation of programmed death 1 on T cells and natural killer cells, thereby inhibiting their activation and anti-cancer function. Inhibition of GFAT in IFNγ-treated cancer cells was found to inhibit PD-L1 glycosylation, accelerating its proteasomal degradation and, more importantly, enhancing T cell activation and anti-cancer activity of natural killer cells (62). These findings support the use of GFAT inhibitors to manipulate PD-L1 protein levels and thus improve the efficacy of immunotherapy for lung cancer.

### Other Intermediates in Gln Pathway

Lysosomal acid lipase (LAL) is an important lipid hydrolase that generates free fatty acids and cholesterol. Ablation of LAL suppresses immune rejection and thus inhibition of LAL induces the growth of human lung cancer cells in mice. Glutamate dehydrogenase (GLUH), which controls the conversion of glutamate to α-ketoglutarate, was also found to be increased in Treg where LAL was inhibited. This suggests that GLUH may be involved in the inhibition of LAL-induced antitumor immunity (63). GLUH may also be involved in the developing process of drug resistance. Hypoxia increases Gln uptake, glutamate to α-ketoglutarate flux and ATP production in lung cancer cells through upregulation of GLUH expression. Increased GLUH expression inhibited cisplatin-induced apoptosis and caused colony formation, suggesting that intervention of GLUH could alter the resistance of lung cancer cells to cisplatin (64). Gln synthetase (GS) is responsible for the *de novo* Gln synthesis by catalyzing the condensation of glutamate and ammonia, and simultaneously GS is necessary for cellular adaptation to Gln deprivation. Ectopic overexpression of RHPN2 promotes *c-Myc* protein stability by phosphorylating the Ser62 site and increasing the expression of *c-Myc* targeted Gln synthetase, which in turn confers resistance of lung cancer cells to Gln depletion. Analysis of GS expression in clinical samples showed that GS expression was elevated in tumor cells and that high levels of GS were significantly associated with poorer overall survival time in lung adenocarcinoma patients (65). L-Phosphinothricin (glufosinate or 2-amino-4-((hydroxy(methyl) phosphinyl) butyric acid ammonium salt (AHPB) is a structural analogue of glutamate and is a recognized herbicide that takes effects on weeds by inhibiting Gln synthetase. Phosphinothricin’s cytotoxic activity against breast and lung cancer cells was detected by Tamer M. Sakr et al. suggesting that it similarly exerts an effect in lung cancer cells through inhibition of GS (33). Solute carrier family member 7A11 (xCT) is a cystine/glutamate reverse transporter that brings cystine into the cell while exporting glutamate. One cystine molecule can be converted into two cysteine molecules and eventually synthesize glutathione (GSH) that quenches reactive oxygen species (ROS) and maintains redox homeostasis (66). Cancer cells tend to maintain high concentrations of GSH to optimize proper redox homeostasis (67). In non-small cell lung cancer, high xCT expression tends to be associated with more advanced staging and poorer prognosis, therefore, reduced xCT transport activity using sulfasalazine in xCT overexpressing NSCLC cells suppresses cell proliferation and invasion (34). Cheng Zeng et al. found that NOX4 promoted glutaminolysis into total GSH synthesis, and inhibition of GSH production led to significant apoptotic death of NSCLC cells overexpressing NOX4 (68). Therefore, targeting either xCT or NOX4 may affect the cellular redox homeostasis by altering GSH synthesis to deter tumor growth.

## Gln-Assembled Molecules Working as Delivery System

Many human cancer cells, such as lung cancer cells, exhibit oncogene-driven addiction to Gln on account of significantly increased Gln consumption rate in rapidly proliferating cancer cells compared to normal cells. As a result, tumor cells compete with host cells for Gln, leading to an influx of Gln from normal tissue to the tumor. In response to this unique metabolic feature of tumor cells, researchers have developed Gln-derived molecules as carriers to deliver drugs to tumors where they are located, achieving the efficient suppression of tumor cells with less side effects on normal tissues.

The synthetic copolymer polyglutamine is biodegradable and colloidally stable under physiological conditions, which is essential for systemic delivery of drugs or genetic nanocarriers (69). Notably, co-expression of Survivin and MDR1 resulted in strong resistance to chemotherapy treatment. Overcoming drug resistance and enhancing chemotherapy sensitivity is one of the most important features for developing effective cancer therapies (70). It can therefore be hypothesized that polyglutamine-based delivery systems for MDR1/Survivin siRNAs could be used to suppress lung cancer cells that rely heavily on Gln metabolism for survival and growth, especially those with a drug-resistant phenotype. Indeed, *in vivo* and *in vitro* experiments verified that chemical and genetic inhibition of the Gln transporter SLC1A5 reduced the intracellular internalization of the Gln macromolecular analog polyglutamine(PGS)/siRNA complex, suggesting a key role of SLC1A5 in intracellular PGS uptake. Increased uptake of the PGS complex significantly reduced intracellular Gln levels, leading to a moderate reduction in cell growth. To restore drug sensitivity and further enhance antitumor effects, the hybrid siRNA anti-Survivin and anti-MDR1 (siSM) were treated as a model therapeutics *via* a PGS delivery system, resulting in a knockdown of Survivin and MDR1 and further sensitizing cancer cells to the drug cisplatin (DDP), PGS complexes were injected intravenously into a lung *in situ* tumor model, and it was found that PGS/siSM relatively reduced the growth rate of tumors, while simultaneous administration of PGS/siSM and DDP enhanced this effect (35). Jiamin Wu et al. constructed a macromolecular Gln analogue, Gln-functionalized branched polyethylenimine (GPI), as a carrier to deliver anti-CD47 siRNA to block the CD47 “don’t eat me” signal on cancer cells (36). Gln-rich GPI polymorphs similarly utilize the SLC1A5 pathway to selectively facilitate uptake of Gln by Gln-dependent and cisplatin-resistant lung cancer cells, particularly under Gln depletion conditions. GPI further efficiently delivers anti-CD47 siRNA *in vivo* and *in vitro*, down-regulating intratumoral induction of CD47 gene, mRNA and protein functioning as evading phagocytic clearance through binding to the immune receptor SIRPα. Also, probably due to selective delivery, it had no adverse effects on CD47-expressing erythrocytes and platelets. In addition to this, Gln-derived molecules can be used in the diagnosis of lung cancer. Poly(L-γ-Gln-Gln)-paclitaxel (PGG-PTX), as a model polymer, coupled to the T1 contrast agent Gd-DTPA (Gd-diethyltriaminophenylacetic acid) of MRI (PGG-PTX-DTPA-Gd) is used in the delivery system of tumor diagnosis (37). MRI results showed that the signal intensity of PGG-PTX-DTPA-GdNPs was significantly enhanced and prolonged in tumor tissues compared to free Gd-DTPA, which may be associated with increased reflexivity and tumor accumulation. A registered clinical trial (NCT01697930) has been conducted applying positron emission tomography (PET) to validate the tumor imaging characteristics of fluorine 18-(2S,4R)-4-fluoroglutamine (FGln), a glutamine analog radiologic imaging agent. This trial included a lung cancer patient detected with KRAS mutation known for regulatory roles in tumor glutamine utilization and demonstrated significant Tumor Avidity for FGln (71). Above all, drug or gene-editing molecules delivery system based on Gln metabolism characteristics is a new therapeutic strategy that may be able to benefit lung cancer patients in the future.

## Reversing Treatment Tolerance

Currently, in addition to surgical treatment, treatment strategies such as chemotherapy, radiotherapy and targeted therapy can be used as replacement options for lung cancer patients. However, with the use of drugs, many patients develop tolerance to these treatment regimens, which reduces the efficacy. Researchers have found that Gln metabolism plays a critical role in the mechanisms of tolerance generation. Platinum resistance is usually accompanied by alterations in a number of metabolic pathways, such as central carbon metabolism, the tricarboxylic acid cycle, and Gln metabolism. Therefore, it’s predictable that the combination of glutaminase inhibitors and chemotherapy may own the potential on re-sensitizing paclitaxel and cisplatin-resistant cancer cell lines to chemotherapy (72, 73). Cisplatin is the most widely used chemotherapeutic agent, and resistance to this cyclotoxic agent in cancer patients is a major problem in clinical treatment for cancer patients. Both Gln and glutamate showed a decreasing trend in cisplatin-resistant lung cancer cells, while Gln was one of the most significantly altered metabolites in cisplatin-treated A549 xenograft mice, suggesting that Gln metabolism makes a great difference in cisplatin chemotherapy resistance in lung cancer (74). Florine Obrist et al. found that cisplatin-resistant cells have a particularly strong dependence on Gln. Thus, Gln depletion was sufficient to restore the response of initially cisplatin-resistant cell line to cisplatin, and conversely, supplementation with Gln rescues cisplatin-resistant clones from starvation-induced death (75). In a study by Gunnar Boysen et al, GLS inhibition elevated tumor response to radiotherapy by ~30%, suggesting that Gln is of great importance in protecting tumor cells from radiation-induced injury (76). In subsequent experiments with mouse xenografts, short-term CB-839 treatment reduced serum glutathione levels by at least 50%, while tumor xenografts showed a 30% increase in response to radiotherapy. The above results suggest that targeting Gln metabolism for reversal of chemoresistance can also be applied as a radiosensitizer (77).

The emergence of secondary drug resistance is the main reason for the failure of epidermal growth factor receptor tyrosine kinase inhibitors (EGFR-TKIs) as targeted therapy for non-small cell lung cancer (78). *EGFR* mutations in NSCLC cells significantly increase the expression of the Gln transporter(SLC1A5), which increases Gln input. Intermediates from Gln pathway activate *EGFR* downstream signals, including mTOR, ERK1/2, and STAT3, which is an important reason for the reduced sensitivity of NSCLC to EGFR-TKIs (79, 80). Induction of Gln deficiency or SLC1A5 inhibition/silencing inhibited NSCLC cell proliferation and reduced cellular uptake of Gln. Thus, the combination of SLC1A5 inhibitor V9302 and Almonertinib (an EGFR-TKI) had a synergistic inhibitory effect on NSCLC proliferation (81). In a study by Meng Xia et al, we found that Gln utilization was higher in *KRAS* mutant NSCLC than in *KRAS* wild type. Dual inhibition of the MEK-ERK pathway (classical tumor signaling pathway) and Gln metabolism can induce redox stress, as evidenced by reduced mitochondrial membrane potential, elevated ROS levels, and inhibition of Gln degradation, and may be an effective therapeutic strategy for *KRAS*-driven NSCLC (82). Gln related metabolite levels differed significantly between gefitinib-resistant and gefitinib-sensitive cells, with glutamate ammonia ligase (GLUL) playing an important role in determining the sensitivity of non-small cell lung cancer to gefitinib. Elevated levels of Gln synthetase encoded by glutamate-ammonia ligase mediates the increase in Gln anabolism and subsequently sensitizes NSCLC to gefitinib (83). In addition, gefitinib was able to alter the functional activity of nutrient and drug transport systems in lung cancer cells by inhibiting breast cancer resistance protein, but specifically increasing the uptake of glucose and Gln (84). Thus, inhibition of Gln utilization could be a potential therapeutic strategy to overcome gefitinib resistance clinically. With known sensitivity of s-phase cells to rapamycin, *KRAS*-driven cancer cells bypass the Gln-dependent G1 cell cycle checkpoint and are arrested in s-phase when Gln utilization is inhibited. Thus, interference with Gln utilization sensitized K-Ras mutant cancer cells to the apoptotic effects of rapamycin (85). All of the above findings suggest that Gln metabolism is involved in the pathway of resistance generation in many lung cancer patients, providing us with an entry point for reversing drug resistance.

## Relieving Complications Through Altering Gln Intake

Patients with lung cancer undergoing radiotherapy may induce complications such as acute radiation-induced esophagitis (RIE), which can lead to a significant increase in mortality as well as delayed treatment outcomes. Certain drugs may be effective in preventing or at least reducing the incidence or severity of complications during radiotherapy for lung cancer. Gln, a drug with potential radioprotective properties, is the main oxidizing raw material for intestinal epithelial cells and is required to maintain their structural integrity. Also, it is a precursor of nucleotides required for cell regeneration and a source of glutathione, a potent antioxidant (86). Thus, the benefits of exogenous Gln have been evaluated in clinical trials and models of different respiratory diseases (87). Although Gln is continuously provided by skeletal muscle in the high catabolic state of cancer, the significantly elevated Gln depletion will not be overcome by increased synthesis over time (88). This leads to impaired acid-base equilibrium, immune function and epithelial integrity of the gut (89). Erkan Topkan et al. administered Gln supplementation to patients with stage IIIB NSCLC undergoing radiotherapy and found no significant negative effects on tumor control or survival outcomes. In addition, Gln not only reduced the severity and incidence of acute and late radiotherapy-associated esophagitis, but also appeared to have a beneficial effect in preventing weight loss and unplanned treatment delays (89, 90). Manuel algara et al. similarly evaluated the effectiveness of oral Gln intake in preventing radiotherapy-induced esophagitis in lung cancer patients and found that oral Gln intake may have an important role in preventing esophageal complications accompanying radiotherapy in lung cancer patients, but randomized trials are needed to confirm this effect (91). A study by Kanyilmaz Gul et al. showed that oral Gln intake exhibited a preventive and/or delaying effect on radiation esophagitis in terms of both esophageal transit time and serum immune parameters (92). However, other investigators have also found that modulating Gln intake in patients with lung cancer may result in different outcomes. Jennifer Pascoe et al. evaluated Beta-hydroxy beta-methylbutyrate/arginine/glutamine (HMB/Arg/Gln) in newly diagnosed advanced lung cancer patients, but ultimately found that HMB/Arg/Gln reduced the odds of treatment success, contrary to the hypothesis (93). Gln did not demonstrate a corresponding effect on radiotherapy-induced esophagitis in the study by Abdul Rahman Jazieh et al. consistently, which may be due to the specific combination of chemotherapeutic agents or inappropriate doses of Gln (86). In a study by Katrine S. Pedersen et al, restricting Gln intake significantly attenuated the growth of two homozygous mouse tumor models of breast and lung cancer, both pharmacologically and physiologically, and reduced markers of muscle atrophy signaling in hormonal mice. Interestingly, further experiments revealed that wheel exercise eliminated tumor-induced upregulation of the muscle Gln transporter and myogenic inhibitor signaling pathways. Also, wheel exercise completely eliminated tumor-induced weight loss and lean body mass, independent of the effect of wheel exercise on tumor growth (94). In summary, whether altering Gln intake affects survival in lung cancer patients is still unknown and requiring further experimental validation.

## Discussion

Metabolic reprograming occupies an irreplaceable role in tumor cells and has become a hot topic in recent years for researchers attempting to develop new tumor interventions. Gln is the most abundant amino acid in the human body, and it not only provides essential energy and biosynthetic raw materials for tumor cells, but is also an important substance through which tumor cells maintain redox homeostasis in the tumor microenvironment. It was recognized that intermediates from Gln pathway are significantly altered in lung cancer patients and can be used as assisted diagnostic and prognostic factor in lung cancer patients, suggesting its involvement in the tumorigenesis of lung cancer as well. In this review, we describe that the proliferation and invasion of lung cancer cells can be inhibited by directly targeting molecules in the Gln pathway such as Gln transporters, glutaminase, GFAT, xCT, or enzymes involved in biosynthesis or redox homeostasis. Moreover, glutamine dependency is very common in cancer patients, and Gln assembled molecules can be exploited as carriers, delivering drugs/gene-editing molecules with anti-tumor effects directly to the tumor location. For lung cancer patients using adjuvant therapy, the application through targeting glutamine metabolic pathway can enhance the sensitivity to treatment, alleviate complications and ultimately improve survival time and survival quality of lung cancer patients.

Among these existing studies, we can affirm that Gln has a non-negligible role in the development of lung cancer cells. By intervening in the Gln metabolic pathway, we can gain plentiful inspiration for the progression of lung cancer diagnosis and treatment strategies. Nevertheless, the practical application of Gln metabolism in the treatment of lung cancer patients is still very limited, and further efforts are needed to bring benefits to the lung cancer patient population. Meanwhile, metabolic remodeling in cancer cells exists in a variety of pathways, and while most studies have focused on altered glucose and Gln metabolism, cancer cells also utilize other substantial nutrients, including the sulfur-containing amino acids cysteine, methionine, essential fatty acids, choline, trace metals, and vitamins. Future studies investigating the altered pathways of these substances in the tumor microenvironment will certainly yield more potential therapeutic targets that can be applied in lung cancer patients.

## Author Contributions

Conceptualization, GX and ET. Methodology, ET. Investigation, ET. Resources, ET and SL. Writing—original draft preparation, ET, SL, ZZ, and RZ. Writing—review and editing, DH, TG and TZ. Visualization, ET and SL. Supervision, GX. All authors listed have made substantial, direct, and intellectual contribution to the work and approved it for publication.

## Conflict of Interest

The authors declare that the research was conducted in the absence of any commercial or financial relationships that could be construed as a potential conflict of interest.

## Publisher’s Note

All claims expressed in this article are solely those of the authors and do not necessarily represent those of their affiliated organizations, or those of the publisher, the editors and the reviewers. Any product that may be evaluated in this article, or claim that may be made by its manufacturer, is not guaranteed or endorsed by the publisher.
